# Pleomorphic adenoma of nasal septum, an unusual site: a case report from Nepal

**DOI:** 10.1093/omcr/omac152

**Published:** 2023-01-18

**Authors:** Gyan Raj Aryal, Nischal Shrestha, Meenakshi Basnet, Oshan Shrestha, Pratikshya Sharma

**Affiliations:** Department of Otorhinolaryngology-Head and Neck Surgery, Nobel Medical College Teaching Hospital, Biratnagar, Nepal; Department of Internal Medicine, Nobel Medical College Teaching Hospital, Biratnagar, Nepal; Department of Otorhinolaryngology-Head and Neck Surgery, Nobel Medical College Teaching Hospital, Biratnagar, Nepal; Department of Pathology, Nobel Medical College Teaching Hospital, Biratnagar, Nepal; Department of Internal Medicine, Nobel Medical College Teaching Hospital, Biratnagar, Nepal

## Abstract

Pleomorphic adenoma is the commonest benign salivary glands tumor. Major salivary glands are mainly involved, and there is very low reported incidence of such tumor in minor salivary glands of nasal cavity. We report a case of 69-year-old woman who complaint of chronic left nasal obstruction and recurrent scanty bleeding from left nostril for last 2 months. On anterior Rhinoscopy examination, there was a pinkish non-tender mass in left nasal cavity arising from cartilaginous part of nasal septum. Intranasal endoscopic excision was done under general anesthesia and histopathological examination of excised tissue revealed pleomorphic adenoma. Patient was discharged after 3 days. There was no recurrence at sixth month of follow-up.

## INTRODUCTION

Pleomorphic adenoma (PA) is the commonest benign salivary gland tumor, which can occur at any locations wherever salivary gland tissue is found. It mostly involves the major salivary glands with highest incidence in the parotid gland [1]. The minor salivary glands involvement is only about 8%, which includes the nasal cavity, paranasal sinuses, hypopharynx, pharynx, larynx, trachea and lacrimal glands [1]. And among them, only 0.4% incidence in the nasal cavity has been reported [2]. These tumors have their peak incidence in third-sixth decade and have been observed more in females [1].

PA in nasal cavity are often misidentified because of their high cellularity and less stromal component, contrast to those found elsewhere [3, 4]. They are slow growing rare tumor, which are liable to recur even after complete excision [5].

## CASE REPORT

We report a case of a 69-year-old Nepalese woman who presented to outpatient department of Otorhinolaryngology with complaint of chronic left nasal obstruction and occasional recurrent scanty bleeding from left nostril for the last 2 months. There was no history of excessive sneezing, nose trauma, prior surgery, weight loss, fever or facial pain. She was non-smoker, did not drink alcohol and denied any history of substance abuse. On anterior Rhinoscopy examination, there was pinkish polypoidal, non-tender nasal mass in the left nasal cavity. The probe could be passed around the mass except in its medial aspect, where it was attached to the septum. Mild bleeding on probing was seen. Neck lymph nodes were impalpable. All cranial nerve examination was also normal.

**Figure 1:**
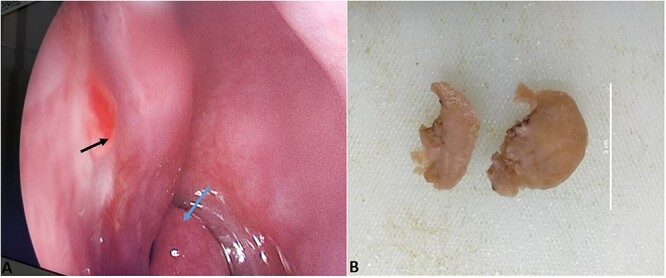
(A) Pleomorphic adenoma in left nasal septum (black arrow); left middle turbinate (blue arrow). (B) Gross picture of excised mass from left nasal septum.

With clinical diagnosis of bleeding granuloma endoscopic excisional biopsy was done under general anesthesia. Smooth, rounded, firm, polypoidal bleeding mass of about 3 × 3 cm^2^ arising from cartilaginous part of nasal septum of left nostril was excised and sent for histopathological examination (Fig. 1).

**Figure 2:**
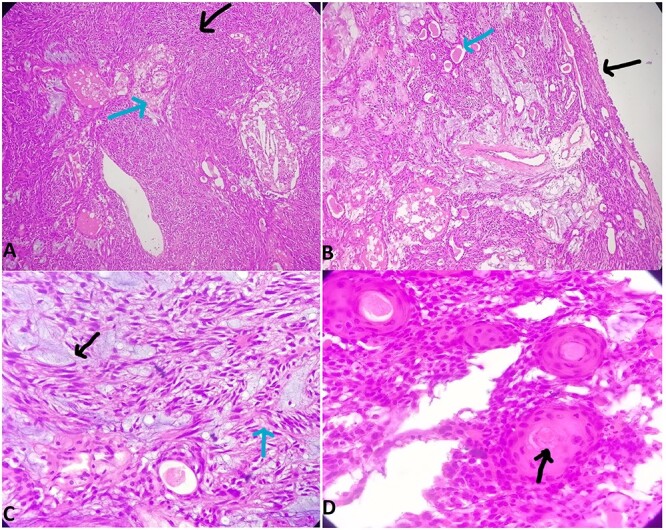
Histology section using hematoxylin and eosin staining. (A) Cellular tumor arranged in sheets (black arrow); sebaceous differentiation (blue arrow). (B) Intact respiratory lining epithelium (black arrow); tubules containing eosinophilic acellular secretion (blue arrow). (C) Areas of myxoid matrix (black arrow); myoepithelial cells melting in myxoid stroma (blue arrow). (D) Squamous differentiation.

Microscopy revealed a well-circumscribed cellular tumor comprising both epithelial and myoepithelial cells. The tumor cells were arranged predominantly in sheets, focally in tubules and trabeculae with an overlying intact respiratory lining epithelium (Fig. 2A and B). The tumor cells lining the tubules in the abluminal layer exhibit myoepithelial differentiation with plasmacytoid, spindled as well as epithelioid morphology and were relatively monomorphic. Most of these tubules contain eosinophilic acellular secretions in their lumen. Multiple areas of myxoid matrix are identified with the myoepithelial cells seen to be melting in the myxoid stroma (Fig. 2C). Focal areas showed tumor cells arranged in cribriform pattern. Some areas of abrupt squamous differentiation and sebaceous differentiation were also identified (Fig. 2D and A). Morphological features were consistent with cellular pleomorphic adenoma.

Patient was discharged after 3 days. There was no sign of recurrence at sixth month of follow-up.

## DISCUSSION

Intranasal PA, a rare finding, was first reported back in 1929 [6]. Though unusual, 40 case studies of nasal cavity PA by Compagno and Wong (1997) and 59 cases by Wakami (1996) have been reported [6]. Literature review of 101 cases of sinonasal PA by Rha et al. (2018) showed the incidence more common in females. Most common site of origin of nasal PA was septum (57.42%) with one-sided nasal blockage as the most common presenting symptom followed by epistaxis, mass in the nose, external swelling, epiphora and mucopurulent rhinorrhea [7].

Diagnosis of nasal cavity PA is confirmed by histopathological examination. Histologically, PA comprises of epithelial and myoepithelial cells in stromal matrix. However, in PA of nasal cavity, myoepithelial cellularity is unusually increased, which makes diagnosis difficult sometimes as revealed by Compagno and Wong [3] as well as Shih-Hung [4]. A misdiagnosed case of nasal PA as adenoid cystic carcinoma was reported by Haberman and Stanely (1989) [8]. Computed tomography scan and magnetic resonance imaging helps in locating the tumor rather than diagnosing.

Although benign, it can have local recurrences. The mesenchymal component myxoid stroma could be locus for future recurrence if it gets spilled into the surgical field [9]. Occasionally, it can behave like malignant tumor. About, 2.5–10% malignant change over rate with female majority was reported by Compango et al. reported [3].

Wide local resection with histological clear margin is generally performed for intranasal pleomorphic adenoma to reduce the rate of recurrence [1]. Different surgical approaches like intranasal endoscopic, transnasal endoscopic, lateral rhinotomy, external rhinoplasty and mid-facial degloving can be used [1].

In conclusion, nasal PA is not only rare tumor, but also difficult to diagnose. PA has high myoepithelial cellularity with less stromal matrix. In case of chronic unilateral nasal blockage due to mass, PA should be considered as a differential diagnosis. The recurrences of PA over a duration of 7–25 years after primary surgery have been reported [10]. Thus, long-term follow-up is necessary as it gives an idea of extent of recurrence, if any, or malignant change over as well as the feasibility of treatment.
